# Integrating a Soft Pneumatic Gripper in a Robotic System for High-Speed Stable Handling of Raw Oysters

**DOI:** 10.3390/foods14162875

**Published:** 2025-08-19

**Authors:** Yang Zhang, Zhongkui Wang

**Affiliations:** 1 Graduate School of Science and Engineering, Ritsumeikan University, Kusatsu 525-8577, Japan; gr0607xp@ed.ritsumei.ac.jp; 2 Department of Robotics, Ritsumeikan University, Kusatsu 525-8577, Japan

**Keywords:** robotic system, soft gripper, stable, high-speed handling, aquatic product, raw oyster, oyster orientation, optimal actuation pressure

## Abstract

Pick-and-place handling of aquatic products (e.g., raw oyster) in packing processing remains manual, despite advances in soft robotic grippers as well as robotic systems that offer a path to automation in food production lines. In this study, we focused on the automation of raw-oyster handling which can be achieved by a robotic system equipped with a soft robotic gripper. However, raw oysters are fragile and prone to large damage during robotic handling, while high-speed handling generates inertial effects. Minimizing the grasping force is thus essential to protect raw oysters, while preserving the grasping stability is required. To address, this study introduces and validates a robotic system equipped with a soft pneumatic gripper for raw-oyster handling task in food production lines. Finite element analysis (FEA) was employed to discuss the effect of gripper actuation pressure on finger deflection and gripper grasping force, revealing a trade-off: increasing actuation pressure improves stability but raises grasping force, whereas reducing actuation pressure causes excessive swing and tossing problems. An optimal actuation pressure of the soft gripper was identified as grasping stability and oyster integrity, minimizing swing while preventing excessive grasping force. Handling performance of the robotic system was experimentally evaluated with raw oysters under different actuation pressures and oyster orientations. Under the optimal actuation pressure confirmed in FEA, the robotic system achieved a handling success rate of 100% (15/15) without obvious misalignment and large damage of raw oysters, which confirmed its adaptability for high-speed, stable handling. This study offers a reference of robotic systems for handling fragile aquatic products and indicates that optimal actuation pressure can protect such products during robotic handling, thereby facilitating the automation of aquatic product processing.

## 1. Introduction

Aquatic products are essential for meeting global food demand [1]. Oysters are an important category of aquatic products, offering high nutritional value with comparatively low environmental impact [2,3]. Global oyster farming yields 7.1 million tons [4]. Japan is one of the main oyster producers, with production areas such as Hiroshima and Miyagi Prefectures [5,6]. Raw oysters are regarded as a premium product [7]. The processing of such fresh products includes peeling, cleaning, grading, and packaging, as they are highly perishable after harvest [8]. Pick-and-place handling during packaging directly influences product uniformity and production efficiency [9]. Currently, the handling of raw oysters remains largely manual [10]. Workers pick raw oysters from conveyor belts and place them into plastic trays [11]. Due to the wet, slippery surface and soft, fragile tissue, workers handle raw oysters gently and carefully to preserve their integrity [12].

Manual handling of raw oysters disrupts cold-chain continuity [13]. It also increases the risk of microbial and viral contamination [14,15], while repetitive, heavy work increases the incidence of human errors [16]. These factors accelerate spoilage and degrade product quality. Concurrent population aging and labor shortages, especially in Japan, erode seafood processing capacity and threaten sector sustainability [17,18]. Therefore, deploying robotic systems with grippers for labor-intensive oyster handling is essential [19]. It enables full cold-chain automation and enhances market competitiveness [20,21,22].

However, automation of high-speed, stable handling of raw oysters remains limited [23]. Their softness and fragility make them susceptible to damage under large grasping force [24]. For raw oysters, successful robotic handling is considered as the absence of tossing, obvious misalignment on the tray, or large damage. Soft robotic grippers, by exploiting compliance and gentle contact, offer advantages in food handling [25,26]. Soft pneumatic grippers are particularly attractive for their low mass, fast response, and ease of implementation [27,28]. Nonetheless, the precise control of grasping force at high speeds remains difficult. Insufficient grasping force can cause swinging or tossing, whereas excessive force may result in large damage [29,30,31]. In pneumatic soft grippers, actuation pressure directly influences grasping force, thereby influencing handling success [32]. Material nonlinearity and hysteresis of soft grippers further limit precise force control [33,34,35]. In addition, conveyor belt types and actuation pressures also may affect placement accuracy. Under high-speed handling, the relationship between actuation pressure and oyster handling success remains insufficiently discussed. Consequently, the high-speed, stable pick-and-place handling of raw oysters requires considerations distinct from those for conventional food products [36,37]. Gentle yet efficient automated handling by robotic systems has become pivotal for industrial upgrading [38,39,40].

**Figure 1:**
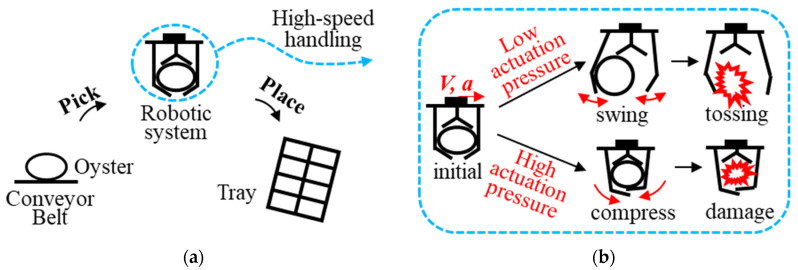
An overview of this work: (**a**) a demonstration of the pick-and-place handling of raw oysters; (**b**) illustration of the effect of different actuation pressures of the soft gripper.

To address these challenges, we focus on integrating a soft pneumatic gripper in a robotic system for the high-speed, stable handling of raw oysters. Figure 1a presents the robotic automation workflow, while Figure 1b illustrates potential handling issues under varying actuation pressures. The main contributions are as follows: (1) a robotic system integrating a soft pneumatic gripper and related subsystems is provided for the high-speed, stable handling of raw oysters; (2) finite element analysis is conducted to compare finger deflection and gripper grasping force during motion under different actuation pressures; (3) raw-oyster experiments are conducted to evaluate the handling performance of the robotic system, and the effects of actuation pressure and oyster orientation. The rest of this paper comprises Section 2 of the materials and methods, Section 3 of results, Section 4 of the discussion, and Section 5 of the conclusion.

## 2. Materials and Methods

### 2.1. Configuration of Robotic System for Raw-Oyster Handling

#### 2.1.1. Robotic System

**Figure 2:**
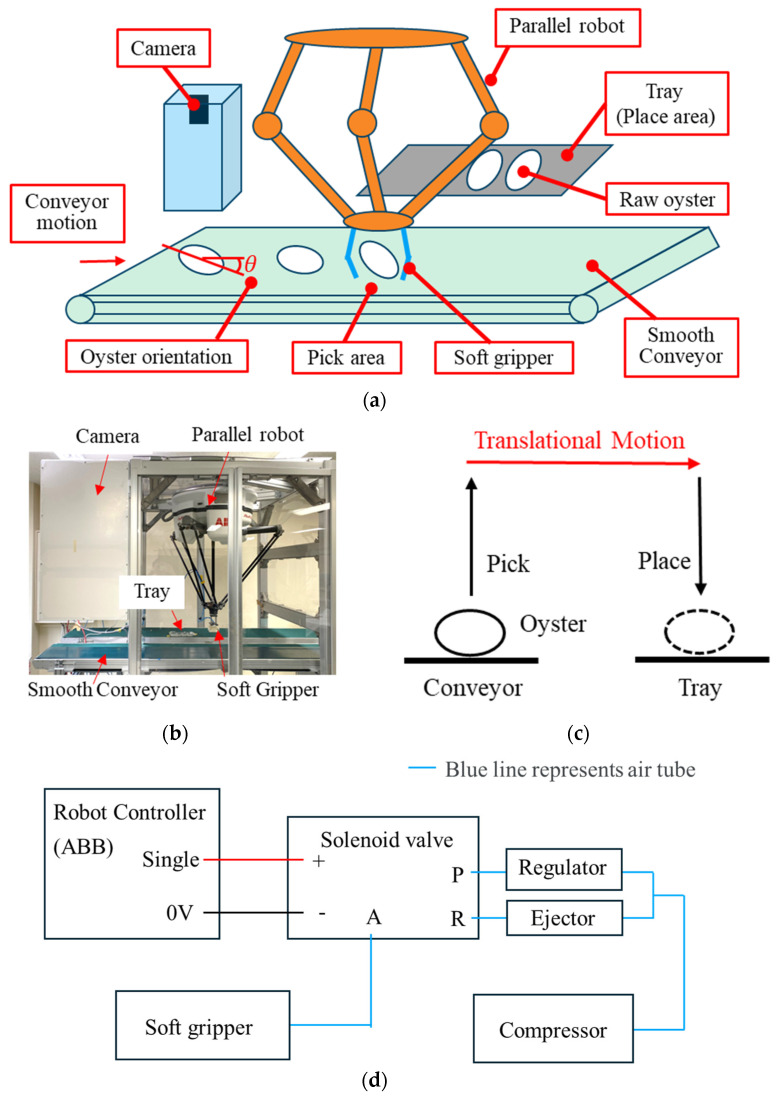
Robotic system for raw-oyster handling: (**a**) 3D schematic of robotic setup; (**b**) photograph of the actual system; (**c**) schematic of the gripper trajectories; (**d**) schematic of the pneumatic system.

Figure 2a provides a robotic system for raw-oyster handling, which comprises a soft pneumatic gripper (designed by us), a parallel robot (IRB 360 FlexPicker, ABB, Zurich, Switzerland), a camera system (scA1300-32gc, Basler, Ahrensburg, Germany), a pneumatic system (designed by us), and a conveyor system with a smooth belt (improved by us). The camera detects oysters on the moving belt at a preset speed, and the gripper extracts each oyster and transfers it to the place area of the tray. Figure 2b shows the actual system.

Figure 2c illustrates the pick-and-place handling route required to achieve a throughput of 60 oysters per minute. It comprises three phases: pick (from P1 to P2), translational motion (from P2 to P3), and place (from P3 to P4). At the pick position, the gripper closes over 0.05 s to ensure a secure grasp. Following closure, it moves along the place trajectory. The camera measures each oyster’s orientation and computes the rotation angle required. Rotation occurs concurrently with translation and completes before deposition. A placement time of 0.10 s guarantees precise alignment within tray slots. All motion sequences are programmed via ABB’s PickMaster3.55 and RobotStudio6.08 software. The translational motion phase immediately after grasping exerts the greatest influence on product integrity, since it must run at high speed to meet production requirements.

The gripper follows the translational motion after picking an oyster from the conveyor belt, as shown in Figure 2c with a red line. The motion path, which comprises acceleration, constant-speed, and deceleration phases, has an influence on oyster integrity due to inertial effects. Using accelerometer (MPU-9250, ARCELI, Shenzhen, China; sampling rate 300 Hz) to measure acceleration of the parallel robot end during translation motion.

A pneumatic system used to drive the soft gripper is shown in Figure 2d and consists of a compressor, regulator, ejector, and directional control solenoid valve. The positive pressure delivered by the compressor is first adjusted via the regulator, while the vacuum is generated solely by the ejector without passing through the regulator. The regulated positive pressure is connected to port P (supply port) of the solenoid valve, and the negative pressure to port R (exhaust port). By switching the solenoid valve, the appropriate pressure is supplied from port A (output port) to the soft hand. Valve actuation is governed by signals from an ABB robot controller, enabling the fingers’ opening and closing to be precisely synchronized with the robot’s movements.

#### 2.1.2. Soft Pneumatic Gripper

**Figure 3:**
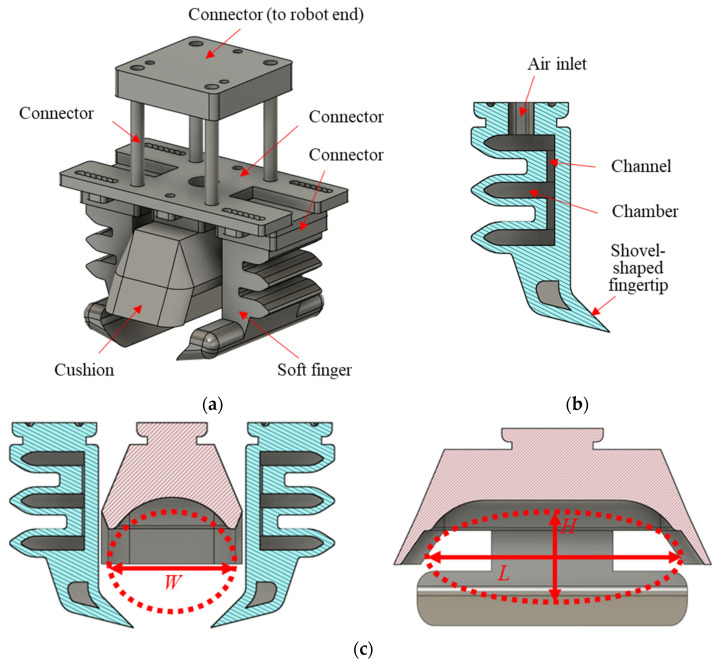
Soft robotic gripper: (**a**) 3D schematic of the soft gripper; (**b**) the section view of soft finger shows the air channels and chambers; (**c**) a containing space in the soft gripper for grasping raw oysters with a size of *L*81.45 mm × *W*43.00 mm × *H*33.35 mm.

Figure 3a illustrates a soft pneumatic gripper design by us, which is described in [41]. It provides a large contact area to minimize raw-oyster deformation and encloses them to restrict motion in all directions without relying solely on friction. The gripper comprises connectors, two soft fingers, and a soft cushion. The connectors secure the cushion, and fingers attach to the robotic end. The soft fingers adopt the multi-chamber PneuNet finger [42] shown in Figure 3b. The pneumatic system controls bending deformation and resistance to inertia through the actuation pressure. Section 3 presents a finite-element analysis of how actuation pressure affects finger deflection and grasping force, and Section 4 validates these findings experimentally.

In Figure 3c, the red dashed region indicates a containing space in the soft gripper, which can wrap and accommodate raw oysters from any direction. The space size can match the oyster average size in Section 4. The CAD model features a shovel-shaped fingertip, corresponding to the oyster length. Horizontal displacement is constrained by the two soft fingers. Vertical motion is controlled by the cushion together with the shovel-shaped fingertips. Front and rear translation is limited by ear-like protrusions on either side of the cushion. This configuration maximizes the contact area between the gripper and product, preventing displacement and slippage during handling.

#### 2.1.3. Conveyor Belt

In this study, the conveyor system for raw oysters is given special consideration. Raw oysters are soft, fragile, and prone to adhering to rough conveyor belts, which increases large damage rates and reduces the success of automatic robotic handling. After comparisons, a smooth-surface belt (SL-M2401N, Bando Chemical Industries, Ltd., Kobe, Japan) is identified as an optimal solution, while the parameters of the smooth conveyor belt are shown in Table 1. The improved conveyor system uses a smooth-surface belt. Its hydrophobic, anti-adhesive surface resists salt-spray corrosion while retaining enough flexibility to prevent shell damage. The belt can be cleaned and replaced with ease, supporting continuous operations and meeting hygiene requirements. It is bonded over the existing coarse conveyor to help the automated handling of raw oysters.

### 2.2. Setup of Finite Element Analysis

Finite element analysis (FEA) were carried out in Abaqus (Dassault Systèmes, Vélizy-Villacoublay, France) [43] to evaluate two key factors. First, the effect of actuation pressures on finger deflection was assessed to confirm whether pressures have influence under acceleration. Second, the effect of driven pressure on finger deflection grasping force during motion was examined to identify any inertial effects.

#### 2.2.1. FEA of Actuation Pressure Effect on Finger Deflection

To perform the FE simulation, we first imported the CAD model of the soft finger into Abaqus and defined the material properties, mesh, and boundary conditions. We considered a soft finger made of a linear elastic material and assigned a Young’s modulus of 2.5 MPa, equivalent to a Shore hardness of A50. The 10-node quadratic tetrahedron hybrid element was used to mesh the soft fingers with a global mesh seed of 3 mm. For interaction definition, the inner and outer surfaces of the finger chambers were considered as self-contact. The contact property was modeled as “hard contact normal behavior” and “penalty tangential behavior” with a friction coefficient of 0.8 (measured in laboratory). The top surface of the soft finger was fixed in space using the boundary condition. The material density was set at 1080 kg/m^3^ for silicone rubber [44].

An explicit solver was used for large deformation. The simulation was conducted in two steps: applying actuation pressure to the inner surfaces of the air chambers and applying an acceleration of 41.19 m/s^2^ to the entire model along the horizontal axis. The value of 41.19 m/s^2^ was selected to represent the extreme load during translational motion, emphasizing the importance of driven pressure. Inertial effects induce the swing of the soft finger, compromising grasp stability.

**Figure 4:**
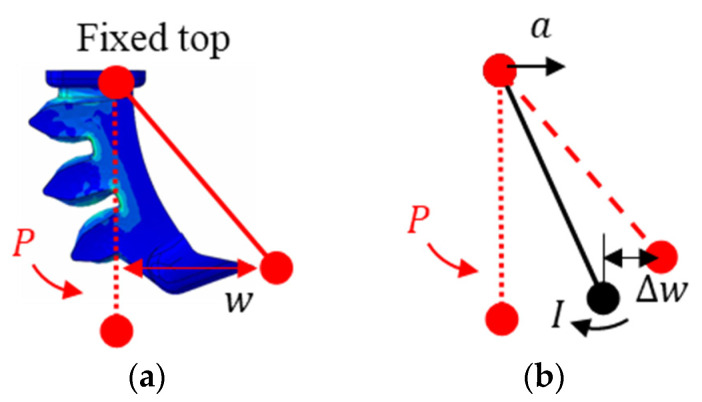
Actuation pressure effect on finger deflection: (**a**) a schematic illustrating the free deformation and deflection of the soft finger under the actuation pressure; (**b**) a schematic illustrating the deflection change of soft finger under inertial loads.

As shown in Figure 4a, the soft finger initially undergoes free deformation under actuation pressure, and its tip displacement corresponds to the deflection w at that pressure. When the same pressure is maintained and an inertial load is applied, the deflection changes by Δw, and the magnitude of this change depends on the actuation pressure and inertial load, as shown in Figure 4b. To compare the maximum swing amplitude, Δw values at an acceleration of 41.19 m/s^2^ were obtained. The deflection variation rate ξ(P) is calculated by Equation (1).

(1) $$\xi (P)=\frac{\Delta w\;(P)}{w(P)}\times 100\%$$

#### 2.2.2. FEA of Actuation Pressure Effect on Grasping Force

**Figure 5:**
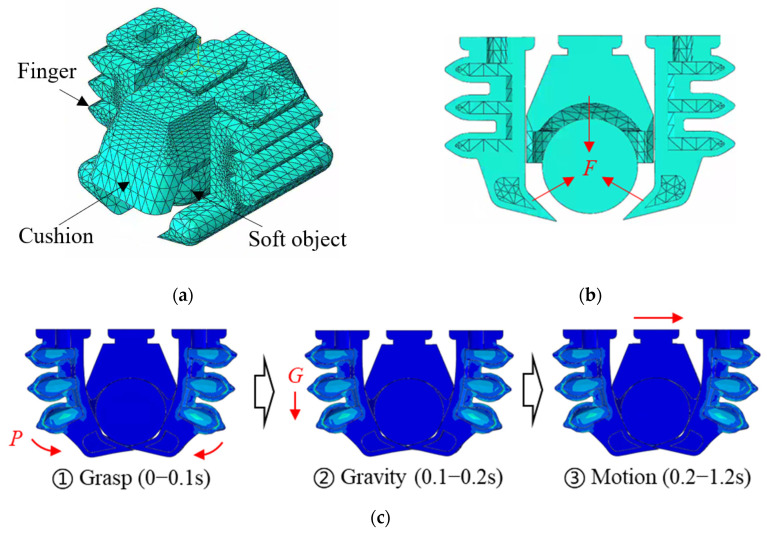
Object handling simulation: (**a**) a gripper model to handle a deformable object; (**b**) a section view to show the grasping force on the object; (**c**) snapshots to show the simulation steps.

The gripper-object model was built for further FEA, as shown in Figure 5a. To align with the translational motion parameters in Section 2.1.1 and the experiments in Section 2.3, the motion parameters in simulations was set the same. The soft object was modeled as capsule shaped like raw oysters, while the Young’s modulus set as 30 kPa, which is close to very soft foods [45,46]. The contact interactions between the soft fingers and the object, and between the center cushion and the object were both set as “surface-to-surface” contact. The contact property was also modeled with “hard contact normal behavior” and “penalty tangential behavior” with a friction coefficient of 0.8 (measured in the laboratory). The boundary constraint was applied to the top surfaces of the fingers and the cushion. Other settings are the same as single-finger simulations. Since object handling relies on the coordinated actions of the cushion and soft fingers, evaluating the effects of actuation pressures necessitates obtaining the grasping force, as shown in Figure 5b.

The simulations were conducted in three steps, as shown in Figure 5c: applying an actuation pressure on the inner surfaces of the air chambers to grasp the object, applying a 1 g “gravity” load to the entire model, and applying motion parameters to the top surfaces of the fingers and the cushion.

#### 2.2.3. Evaluation of Optimal Actuation Pressure

To identify the optimal actuation pressure for the raw-oyster experiments described in Section 2.3, grasping performance was evaluated in terms of peak and average grasping forces. The peak force reflects the maximum force during the interaction between gripper and object, which raises the risk trend of object damage, while the average force indicates the stable grasping of the object. Balancing these two parameters is therefore essential. An evaluation metric, Λ (N), was introduced, as defined in Equation (2), which starts from the average force and incorporates a coefficient term based on the ratio of peak force to average force. The actuation pressure that minimizesΛ is considered optimal.

(2) $$\Lambda =\overset{Average\;force}{\overbrace{\sqrt{\frac{1}{n}\sum_{1}^{n}F_{i}^{2}}}}\overset{Peak\;force}{\;\;\overbrace{(1+\sqrt{\frac{max\{F_{1},F_{2},F_{3},\cdots ,\;F_{n}\}}{\sqrt{\frac{1}{n}\sum_{1}^{n}F_{i}^{2}}}}}})$$

### 2.3. Setup for Raw-Oyster Experiments

**Figure 6:**
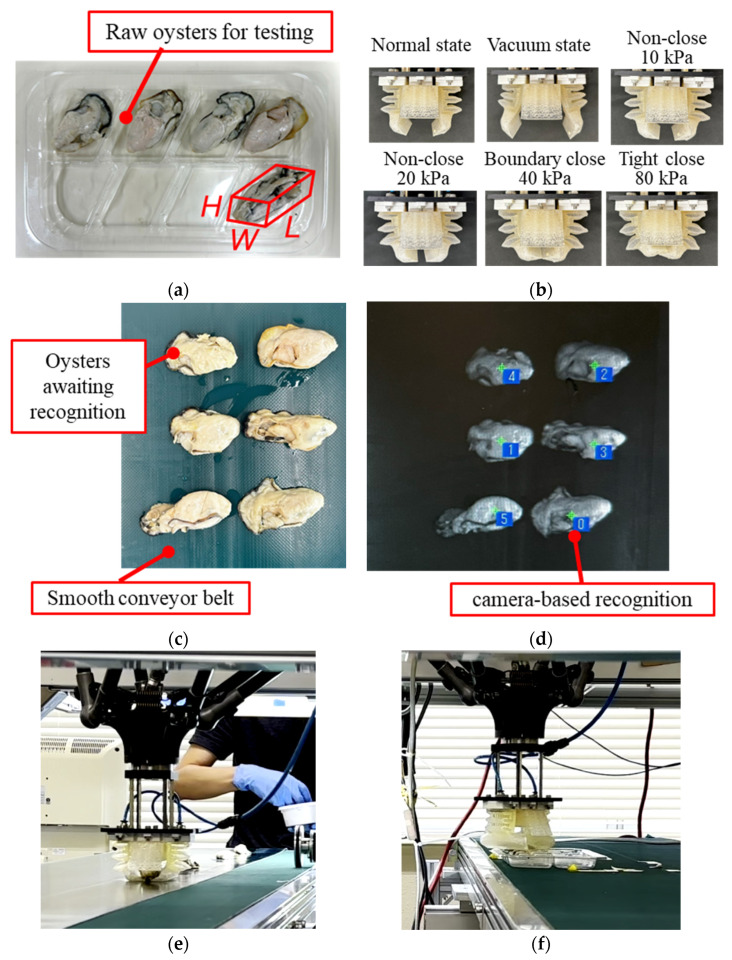
Tested raw oysters and experimental snapshots: (**a**) the raw oysters weighed 13.00 ± 1.05 g with an average size of *L*56.73 ± 3.96 mm × *W*26.19 ± 1.75 mm × *H*17.33 ± 1.54 mm; (**b**) different gripper states; (**c**) raw oysters on the conveyor belt are waiting for recognition; (**d**) the camera-based recognition of raw oysters; (**e**) gripper grasping an oyster from the conveyor belt; (**f**) gripper placing an oyster into the tray.

Standard-specification raw oysters in market were used to evaluate the performance of the robotic system. The tested targets, shown in Figure 6a, weighed 13.00 ± 1.05 g with a size of *L*56.73 ± 3.96 mm × *W*26.19 ± 1.75 mm × *H*17.33 ± 1.54 mm (Appendix A).

This study focuses on the pick-and-place handling operation independent of the upstream automated supply system. Raw oysters were manually fed in various orientations to simulate practical automated-supplying conditions. A plastic tray for packing raw oysters was used in the production line for the handling tests, as shown in Figure 6a. The oysters were manually supplied at three different angles (oyster orientation): approximately 0°, 45°, and 90°. An angle of 0° indicates that the major axis of the oyster aligns with the moving direction of the conveyor belt. During handling, oysters supplied at different angles might require extra rotational motion to align the oysters while placing them in the tray. This extra rotational motion may cause handling failure due to the centrifugal force and slippage. Four actuation pressures (10, 20, 40, and 80 kPa) were applied, producing different gripper states: non-close at 10 and 20 kPa, boundary close at 40 kPa, and tight close at 80 kPa, as shown in Figure 6b.

As shown in Figure 6c,d, oysters were placed on this belt beneath an overhead camera, which captured images of the raw specimens prior to testing. The vision system then determined oyster position on the moving belt, enabling precise deployment of the soft gripper. A smooth belt designed for aquatic-product conveyance minimizes adhesion and residue. Furthermore, at each actuation pressure, tests were conducted at 0°, 45°, and 90° orientations, with five samples per orientation. These angles were selected to represent the sample orientations encountered during transfer from the upstream process to the pick-and-place stage, resulting in 15 tests at each actuation pressure. Figure 6e,f show the moments at which the gripper picked an oyster from the conveyor belt and placed it in the corresponding slot on the plastic tray, respectively.

## 3. Results

### 3.1. Results of Motion Measurement

**Figure 7:**
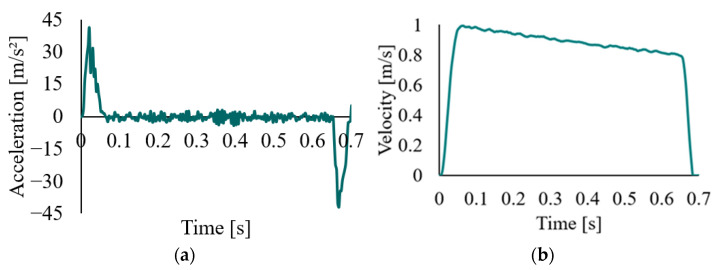
Illustration of translational motion during pick-and-place handling: (**a**) measured data of the robot end acceleration during translational motion; (**b**) velocity data are obtained by integrating the raw acceleration data.

The gripper follows the translational motion after picking an oyster from the conveyor belt, as shown in Figure 2c with a red line. Raw acceleration data are provided in Figure 7a, including a maximum acceleration of 41.19 m/s^2^, and a maximum integrated velocity of 0.99 m/s in Figure 7b. As the robot setting of maximum velocity is 1.00 m/s, the accuracy of the measurement is validated. Errors remain within acceptable and stem from noise introduced by unfiltered raw acceleration data.

### 3.2. Results of Finite Element Analysis

#### 3.2.1. FEA of Actuation Pressure Effect on Finger Deflection

**Figure 8:**
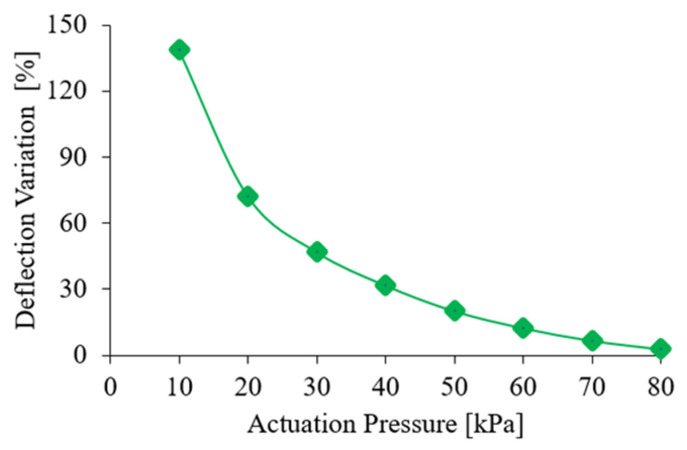
A plot showing soft finger deflection under inertia at different actuation pressures.

Figure 8 shows the variation rate. Table 2 summarizes the related values. Through numerical fitting, the relationship between ξ(P) and *P* can be expressed by Equation (3), with a coefficient of determination of $R^{2}$ = 0.9602. It shows that the soft finger with higher actuation pressure exhibits less swing under the inertial effect.

(3) $$\xi \left(P\right)=0.0376P^{2}-5.0474P+172.6129$$

The results demonstrate that the actuation pressure has a pronounced effect on the soft finger. Under inertial loading, higher actuation pressures reduce swing amplitude, while lower actuation pressures amplify it. Although smaller swings enhance finger stability and promote stable grasping, excessively high actuation pressure increases finger bending and may impose excessive force on the object. Consequently, Section 3.2.2 further investigates the effect of actuation pressure on grasping forces during motion.

#### 3.2.2. FEA of Actuation Pressure Effect on Grasping Force

**Figure 9:**
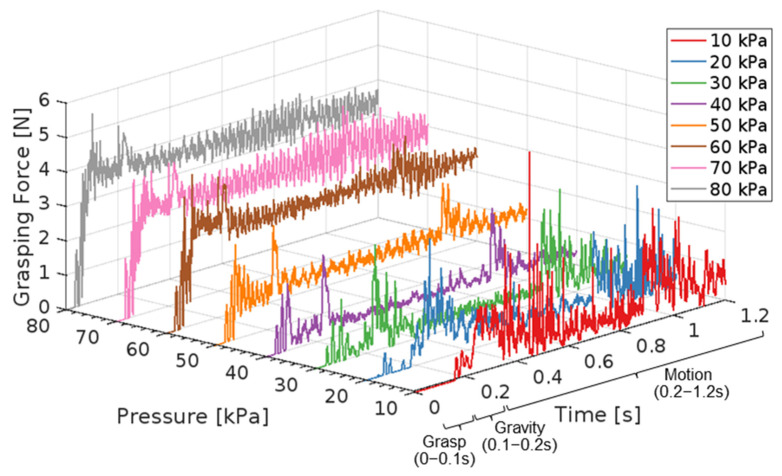
Grasping force on the object surface applied by the soft gripper.

Curves of the grasping forces exerted on the object under varying actuation pressures are presented in Figure 9. The finite element simulations can be observed in Video S1 (Supplementary Materials). During the 0–0.2 s (grasping and gravity steps), the grasping force increased proportionally with the actuation pressure: a higher pressure produced a tighter grip. However, during the subsequent translational motion (after 0.2 s), the relationship between pressure and force became nonlinear. Grasping-force fluctuations closely mirrored the acceleration profile shown in Figure 7a, indicating that inertial effects during acceleration and deceleration produce force peaks corresponding to acceleration peaks. Actuation pressure still exerted a marked influence. At low pressure, the force curve exhibited large oscillations, reflecting unstable grasping in which the object repeatedly collided with both fingers and the central cushion. In contrast, higher pressure yielded a more stable force profile with diminished swing, signifying “tight” clamping throughout the handling. Therefore, the actuation pressure of the soft gripper plays a critical role in grasping force changes under high-speed handling. The peak and average forces under different actuation pressures are provided in Table 3.

Results of grasping forces indicate that neither overly low nor overly high actuation pressures suit the high-speed handling of soft objects. Moderate pressures provide the better compromise between peak and average grasping forces, which achieve stable grasping while protecting the object. Section 3.2.3 identifies an optimal actuation pressure for the soft gripper.

#### 3.2.3. Results of Optimal Actuation Pressure

**Figure 10:**
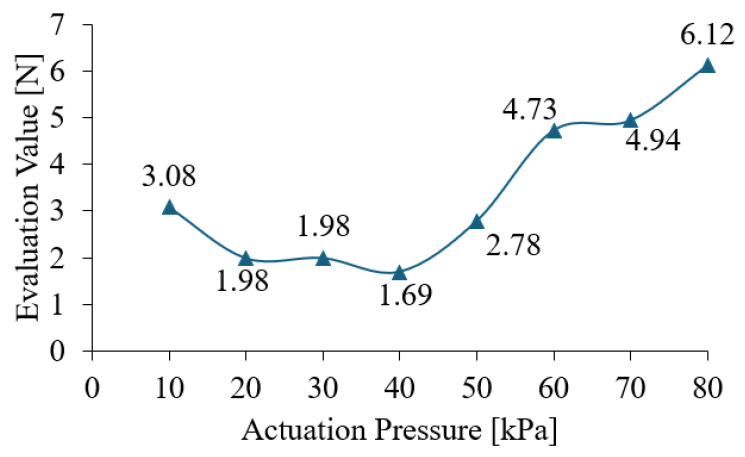
Evaluation result.

Calculated by Equation (2) with grasping forces from FEA in Section 3.2.2, the evaluation results of optimal actuation pressure in Figure 10 show that Λ ranges from 1.69 N to 6.12 N, indicating an optimal actuation pressure exists.

To further discuss the trend that moderate actuation pressure of the soft gripper reduces grasping force compared with too-low or too-high pressures, two more soft objects were simulated additionally. Objects with a Young’s modulus of 60 kPa and 90 kPa were selected, as the modulus of some soft food products is below 100 kPa [47]. As shown in Table 4, both too-low and too-high actuation pressures increase Λ values, owing to excessive swing and “tight” clamping, respectively.

These results of FEA confirm the existence of optimal actuation pressure for soft grippers during handling of soft objects. Under the proposed robotic system and related settings, the optimal actuation pressure is determined to be 40 kPa. Tests of the optimal actuation pressure and performance of the robotic system are conducted in Section 3.3.

### 3.3. Results of Raw Oyster Experiments

#### 3.3.1. Experimental Results of Different Actuation Pressures

To compare the effects of different actuation pressures, tests were conducted with oysters oriented at 0° and processed in batches of five. At the actuation pressure of 40 kPa, all oysters were handled successfully without obvious misalignment and large damage, whereas 10 kPa, 20 kPa and 80 kPa produced low handling success rates. The experiments can be observed in Video S2 (Supplementary Materials).

**Figure 11:**
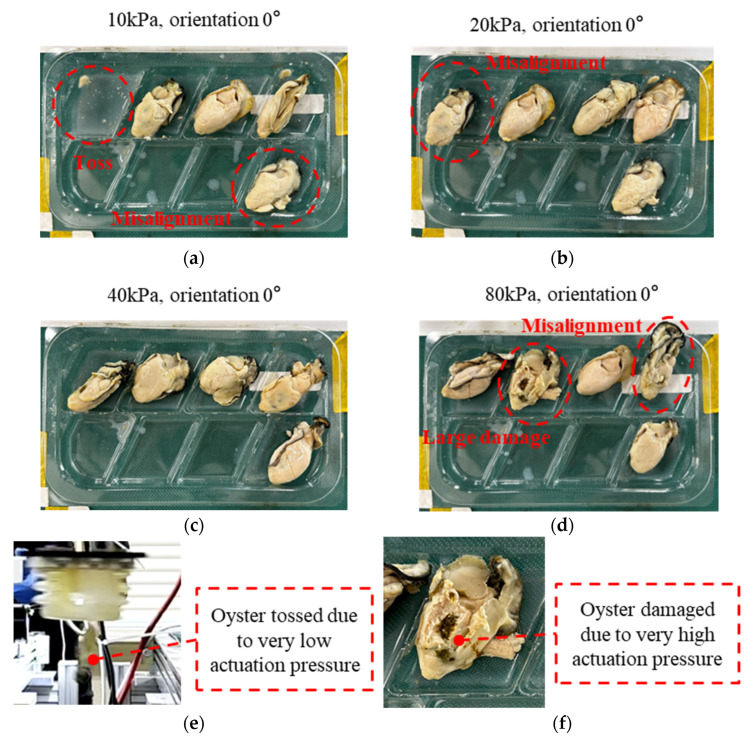
Results of actuation pressure effect: (**a**) a snapshot of the packaged raw oysters under the actuation pressure of 10 kPa; (**b**) a snapshot of the packaged raw oysters under the actuation pressure of 20 kPa; (**c**) a snapshot of the packaged raw oysters under the actuation pressure of 40 kPa; (**d**) a snapshot of the packaged raw oysters under the actuation pressure of 80 kPa; (**e**) a snapshot of oyster tossing; (**f**) a snapshot of the damaged oyster.

The success rate in Table 5 denotes stable and high-speed handling under the optimal actuation pressure of 40 kPa. Handling failures from inappropriate pressure included oyster tossing during handling, obvious misalignment after placing, and large damage, as highlighted in Figure 11a–d. At the actuation pressure of 10 kPa, gripper swings during motion destabilized the grasp and caused oyster tossing, as shown in Figure 11e. At the actuation pressure of 80 kPa, irreversible large damage occurred during handling, as shown in Figure 11f. The actuation pressure of 20 kPa, being closer to the optimal actuation pressure of 40 kPa, improved handling performance but still exhibited obvious misalignment. These results confirm that actuation pressure influences handling performance, validate simulation results, and demonstrate the existence of an optimal actuation pressure of the soft gripper. Under the optimal actuation pressure, the robotic system can achieve a 5/5 handling success rate.

#### 3.3.2. Experimental Results of Different Oyster Orientation

**Figure 12:**
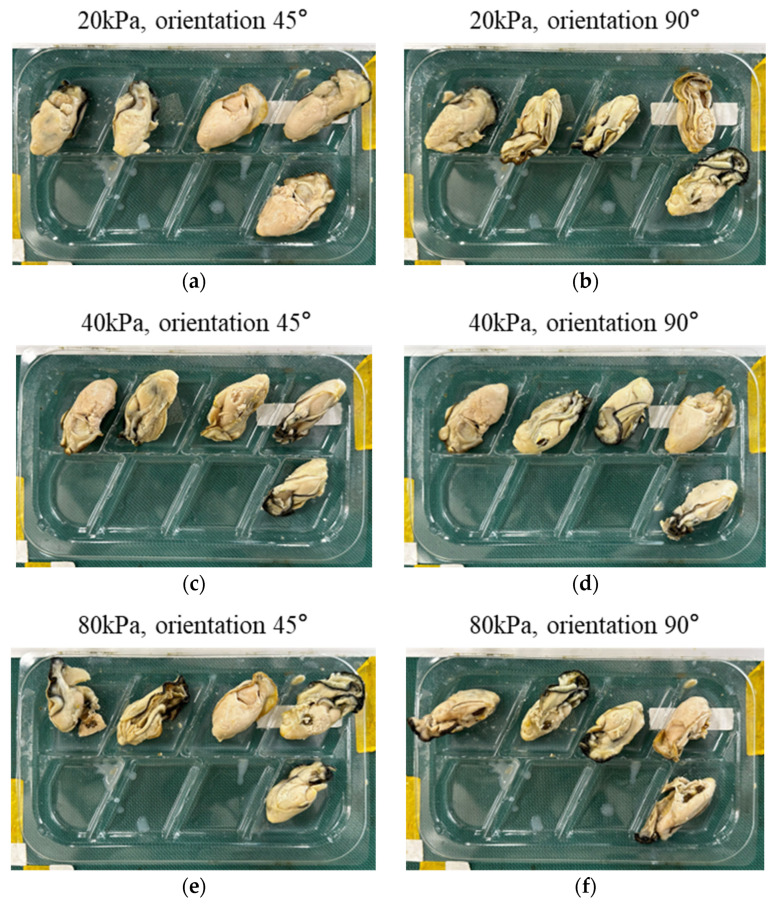
Results of oyster orientation effect: (**a**) a snapshot of the packaged raw oysters actuation pressure of 20 kPa and oyster orientation of 45°; (**b**) a snapshot of the packaged raw oysters actuation pressure of 20 kPa and oyster orientation of 90°; (**c**) a snapshot of the packaged raw oysters actuation pressure of 40 kPa and oyster orientation of 45°; (**d**) a snapshot of the packaged raw oysters actuation pressure of 40 kPa and oyster orientation of 90°; (**e**) a snapshot of the packaged raw oysters actuation pressure of 80 kPa and oyster orientation of 45°; (**f**) a snapshot of the packaged raw oysters actuation pressure of 80 kPa and oyster orientation of 90°.

To compare the influence of oyster orientation, experiments were conducted at actuation pressures of 20 kPa, 40 kPa, and 80 kPa. The unstable grasping observed at 10 kPa is analyzed in Section 3.3.1. The experiments can be observed in Video S2 (Supplementary Materials). Table 6 summarizes the experimental results. At the actuation pressure of 20 kPa, corresponding to the non-close state, obvious misalignment occurred, as shown in Figure 12a,b. At the actuation pressure of 40 kPa, corresponding to the boundary close state, oysters were successfully handled across all tested orientations, as shown in Figure 12c,d. At the actuation pressure of 80 kPa, corresponding to the tight close state, obvious misalignment and large damage occurred, as shown in Figure 12e,f. When oyster orientation is 90°, the soft gripper with raw oysters has rotations around the vertical axis during handling to match the orientation of the tray slot. This rotation amplifies instability and leads to a greater risk of handling failure, yet the actuation pressure of 40 kPa maintained a high handling success rate. The pressures of 20 kPa and 80 kPa have low success rates. Results demonstrate that oyster orientation affects the handling performance of the robotic system, and under optimal actuation pressure, it can also achieve a 10/10 handling success rate without obvious misalignment and large damage of raw oysters.

## 4. Discussion

This study focusses on integrating a soft pneumatic gripper in a robotic system for the high-speed, stable handling of raw oysters, aiming to replace manual pick-and-place handling operations with robotic automation to improve oyster production efficiency and quality. The work is evaluated from three perspectives: robotic system configuration, finite element analysis, and experimental validation.

A high-speed robotic system was introduced, comprising the soft pneumatic gripper, parallel robot with controller, vision subsystem, pneumatic control subsystem, and conveyor subsystem. Beyond generic motion control, the system addressed the specific challenges of raw-oyster handling. The soft gripper employed silicone material to enhance compliance and incorporated a sufficient containing space to maximize the contact area for reducing oyster damage, differing from friction-based grasping designs [11,48]. Given the tissue softness and surface adhesion of oyster, a smooth belt was selected and tested to limit sticking, a consideration essential for handling aquatic products [49]. The robotic system eliminates human–oyster contact, reducing contamination risk, which is critical for food safety. Automation also improves productivity and product consistency, reducing labor costs.

Regarding the finite element analysis, stepwise simulations of pressure effects on the soft finger and gripper revealed influences of actuation pressure on grasping force during high-speed motion. Considering both average and peak grasping forces, the optimal actuation pressure was identified when the simulated soft object exhibited mechanical properties similar to those of oysters. Furthermore, when the Young’s modulus of the simulated object varied, the moderate actuation pressure of the soft gripper still outperformed extreme pressures.

Experiments with raw oysters confirm the existence of optimal actuation pressure. An appropriate actuation pressure achieves high-speed, stable handling without inducing obvious misalignment and large damage, whereas unsuitable pressures can lead to object tossing during motion, misalignment in placing, and large damage. Under optimal pressure, a high handling success rate was achieved. For fragile aquatic products like raw oysters, a moderately loose enveloping grasp outperforms both non-clamping (non-close) and over-clamping (tight close). Experiments further reveal that the orientation of each oyster also influences the handling success. Selecting the appropriate pressure mitigates issues stemming from unfavorable orientations.

The experimental results were consistent with the assumptions in Figure 1b and finite element analysis, indicating that higher soft-finger stiffness does not necessarily yield better performance in high-speed handling. Instead, the soft gripper must have an optimal actuation pressure for the high-speed handling of raw oysters. Future work will extend this optimization by establishing pressure–object relationships and validating the approach across various aquatic products. In addition, adaptive control strategies will be explored to enable rapid adjustment of actuation pressure in response to changes in the motion state when handling.

## 5. Conclusions

This study focuses on integrating a soft pneumatic gripper in a robotic system for the high-speed stable handling of raw oysters. Finite element analysis assessed the effects of actuation pressures on gripper performance. Analysis results show that inertial effects during motion influence finger deflection, with large swings at low pressures. By further evaluating both the peak and mean grasping forces, an actuation pressure of 40 kPa was identified as the optimal pressure for object protection. Raw-oyster experiments tested the handling performance of the robotic system. Experimental results demonstrate that the proposed robotic system could handle raw oysters, and that the optimal actuation pressure enables high-speed, stable handling with a high success rate. Although oyster orientation influenced the handling success rate, the optimal actuation pressure maintained high performance across different tested conditions. In contrast, very low actuation pressure caused oyster tossing, whereas very high pressure resulted in large damage. Obvious misalignment on the tray occurred at both extremes of actuation pressure. This study provides a reference for robotic systems equipped with soft grippers to achieve the high-speed, stable handling of soft, fragile aquatic products such as raw oysters, thereby advancing the automation of aquatic-product processing, particularly in pick-and-place handling. Future work will focus on optimizing actuation pressure and developing adaptive strategies to enhance the adaptability of this robotic system.

## Figures and Tables

**Table 1:** Key parameters of the smooth belt.

Parameter	Material and Value
Shape	Smooth and glossy
Material (front/back)	PU/PET
60 kPa	12.33%
Surface friction coefficient	0.15

**Table 2:** Deflection change rate under different actuation pressures.

Actuation Pressure (kPa)	Deflection Variation Δw (%)
10	138.69
20	72.28
30	46.91
40	31.75
50	20.11
60	12.33
70	6.61
80	2.78

**Table 3:** Peak and average forces of grasping forces under different actuation pressures.

Actuation Pressure (kPa)	Peak Force (N)	Average Force (N)
10	6.01	0.67
20	3.58	0.59
30	3.12	0.50
40	2.46	0.49
50	2.98	1.23
60	4.51	2.47
70	4.31	2.86

**Table 4:** Evaluation results of grasping forces.

Actuation Pressure (kPa)	$\Lambda_{O30kPa}$ (N)	$\Lambda_{O60kPa}$ (N)	$\Lambda_{O90kPa}$ (N)
10	3.08	3.06	5.04
40	1.69	1.76	3.74
80	6.12	5.95	7.83

**Table 5:** Experimental results of the handling tests for raw oysters under different actuation pressures. The circled numbers indicate the reasons for the failed trials: **I**—toss during handling, **II**—misalignment in the tray, and **III**—large damage.

Actuation Pressure (kPa)	Success Rate of Handling
10	3/5 (**I**, **II**)
20	4/5 (**II**)
40	5/5
80	3/5 (**II**, **III**)

**Table 6:** Experimental results of the handling tests for raw oysters under different oyster orientations. The circled numbers indicate the reasons for the failed trials: **I**—toss during handling, **II**—misalignment in the tray, and **III**—large damage.

Actuation Pressure (°)	Oyster Orientation 45°	Oyster Orientation 90°
20	2/5 (**II**)	3/5 (**II**)
40	5/5	5/5
80	3/5 (**II**, **III**)	2/5 (**II**, **III**)

**Figure A1:**
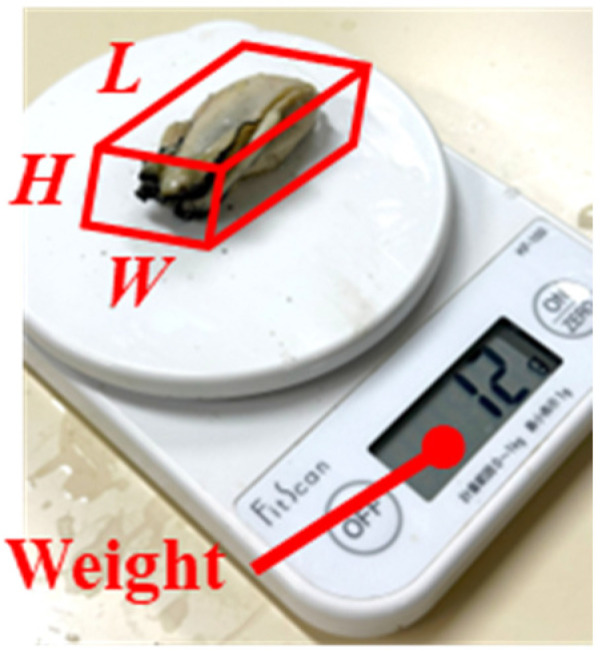
Snapshot of a raw oyster for measurement.

## Data Availability

The original contributions presented in this study are included in the article/Supplementary Material. Further inquiries can be directed to the corresponding author.

## Supplementary Materials

The following supporting information can be downloaded at: https://www.mdpi.com/article/10.3390/foods14162875/s1, Video S1: Finite element analysis, Video S2: Oyster experiments.

## Appendix A

To ensure measurement accuracy, 10 undamaged raw oysters were randomly selected from the same batch and stayed at a room temperature of 27 °C, as shown in Figure A1. Each oyster was weighed on an electronic balance (FitScan, ±1 g accuracy), and the average mass were computed. Length (*L*), width (*W*), and height (*H*) were then measured with a vernier caliper (Sink, ±0.1 mm accuracy). Length was measured along the axis of maximum size, width perpendicular to that axis, and height perpendicular to the length–width plane. Each dimension was measured, and the average value calculated. All measurements were entered sequentially into Table A1 for considering the containing space of soft gripper and experimental tests. Therefore, oysters weighed 13.00 ± 1.05 g with an average size of *L*56.73 ± 3.96 mm × *W*26.19 ± 1.75 mm × *H*17.33 ± 1.54 mm.

**Table A1:** Measurement of raw oysters.

Number	Weight [g]	Length *L* [mm]	Wide *W* [mm]	Height *H* [mm]
1	13	59.5	25.8	16.9
2	12	59.0	23.8	14.6
3	12	59.5	27.9	17.6
4	14	52.2	28.2	19.7
5	15	54.8	29.2	17.0
6	14	61.3	25.2	15.8
7	12	57.5	26.1	16.7
8	13	59.2	25.3	19.5
9	12	48.5	24.2	18.0
10	13	55.8	26.2	17.5
